# Is Experimental Evolution of an Increased Aerobic Exercise Performance in Bank Voles Mediated by Endocannabinoid Signaling Pathway?

**DOI:** 10.3389/fphys.2019.00640

**Published:** 2019-05-28

**Authors:** Ewa Jaromin, Edyta T. Sadowska, Paweł Koteja

**Affiliations:** Institute of Environmental Sciences, Jagiellonian University, Kraków, Poland

**Keywords:** motivation, voluntary exercise, physical activity, endocannabinoids, experimental evolution, selective breeding

## Abstract

The level of physical activity achieved in a given situation depends on both physiological abilities and behavioral characteristics (motivation). We used a unique animal model to test a hypothesis that evolution of an increased aerobic exercise performance can be facilitated by evolution of motivation to undertake physical activity, mediated by brain endocannabinoid system. Bank voles (*Myodes glareolus*) from “aerobic” A lines selected for 22 generations for high swim-induced aerobic metabolism (VO_2_swim) achieved 65% higher “voluntary maximum” VO_2_swim than voles from unselected, “control” C lines. In C lines, VO_2_swim was 24% lower than the maximum forced-running aerobic metabolism (VO_2_run), while in A lines VO_2_swim and VO_2_run were practically the same. Thus, the selection changed both the aerobic capacity and motivation to exercise at the top performance level. We applied a pharmacological treatment manipulation to test a hypothesis that the endocannabinoid signaling pathway 1) affects the voles performance in the aerobic exercise trials, and 2) has been modified in the selection process. Administration of the CB1 receptor antagonist (Rimonabant) did not affect the level of metabolism, but administration of the endocannabinoid reuptake inhibitor (AM404) decreased VO_2_swim both in A and C lines (4%, *p* = 0.03) and tended to decrease VO_2_run (2%, *p* = 0.07). The significant effect of AM404 suggests the involvement of endocannabinoids in signaling pathways controlling the motivation to be active. However, the response to AM404 did not differ between A and C lines (interaction effect, *p* ≥ 0.29). Thus, the results did not provide a support to the hypothesis that modifications of endocannabinoid signaling have played a role in the evolution of increased aerobic exercise performance in our experimental evolution model system.

**Summary Statement:**

The results corroborated involvement of endocannabinoids in the regulation of physical activity, but did not support the hypothesis that modification of endocannabinoid signaling played a role in the evolution of increased aerobic exercise performance in our experimental evolution model.

## Introduction

The level of physical activity achieved by an animal in a given situation depends not only on its physiological and biophysical abilities (e.g., cardiovascular efficiency), but also on motivation to be active, i.e., neurobiological mechanisms. The neurobiological basis of the willingness to be active is a subject of intensive research in both evolutionary biology and medicine. Although both survival and reproductive success depend on behaviors that often require increased performance, not all individuals perform at the upper level of physical abilities (Ekkekakis et al., 2005). As increased physical activity is simultaneously beneficial and risky, animals often choose to perform at much slower pace, far from achieving their physiological limit. Thus, it has been proposed that the neurobiological rewards could motivate animals to undertake intensive physical activity and play a significant role in the evolution of increased physical performance in terrestrial vertebrates, including humans (Ekkekakis et al., 2005; Raichlen et al., 2012). The neurobiological mechanism (motivation) can play a role not only in voluntary physical activity, but also in procedures designed to measure the maximum forced-exercise rate of aerobic metabolism (VO_2_max; Noakes, 2008; Smirmaul et al., 2013). In applied physiology, the measurement of VO_2_max is regarded as a gold standard for evaluation of the overall physical fitness of both top athletes and patients with cardiovascular system diseases (Arena et al., 2010). However, it is controversial if the measured value of VO_2_max does correspond to the real aerobic capacity of an organism (Smirmaul et al., 2013). The details of the neurobiological mechanisms of motivation to exercise at the top performance level as well as of the motivation to undertake any physical activity are particularly important in the context of the prevalence of civilization diseases associated with low physical activity (Bauman et al., 2012; Althoff et al., 2017; Rosenfeld, 2017). Here, we tested a hypothesis that evolution of increased physical performance can be facilitated by evolution of motivation to be active, mediated by eCBs signaling.

eCBs are putatively involved in the regulation of physical activity and in a rewarding feeling after performing strenuous exercise, a phenomenon known as “the runners high” (Dietrich and McDaniel, 2004; Raichlen et al., 2012; Heijnen et al., 2016; Brellenthin et al., 2017; Thompson et al., 2017). An increased level of eCB, especially AEA and 2-AG, was observed in rodents and humans following both aerobic and isometric exercise (Sparing et al., 2003; Heyman et al., 2012; Raichlen et al., 2012; Koltyn et al., 2014; Brellenthin et al., 2017; Crombie et al., 2017). AEA and 2-AG are the ligands of CB1 and CB2 eCB receptors (Kano et al., 2009). CB1 is highly expressed in brain regions that controls movement, emotionality and nociception (De Chiara et al., 2010; Dubreucq et al., 2010, 2013; Gomes da Silva et al., 2010; Raichlen et al., 2013; Tantimonaco et al., 2014; Fuss et al., 2015). Interestingly, Raichlen et al. (2012) demonstrated that the level of AEA in the blood increases after treadmill running in cursorial animals such as humans and dogs, which often engage in endurance physical activity, but not in non-cursorial ferrets. Pharmacological manipulation of eCB system influenced the level of physical activity in rodent models (review in Tantimonaco et al., 2014). Thus, it has been suggested that eCB can motivate high-intensity locomotor behaviors and can be responsible for a variation in locomotor activity across mammals (Raichlen et al., 2012).

The studies of neurophysiological or genetic basis of physical performance are often based on differences between strains of laboratory rodents (e.g., Avila et al., 2017; Kvedaras et al., 2017) and the knowledge concerning neurobiological basis of behavior is severely biased by results from experiments conducted on a few model species (Keifer and Summers, 2016). Here, we used a unique experimental evolution model system: lines of a non-laboratory rodent, the bank vole, from a selection experiment, comprising four replicate lines of voles selected for 1-min-maximum aerobic metabolism achieved during swimming test (VO_2_swim; A – “aerobic” lines) and four lines of unselected, randomly bred voles (C – “control” lines). In generation 21, A-line voles achieved a 58% higher mass-adjusted VO_2_swim than C-line voles (Supplementary Figure S1). However, during the swimming test animals do not necessarily achieve *the maximum* aerobic metabolism, as they can “hang” in water with just the tip of their nose above the water surface. Not surprisingly, in generation 5, VO_2_swim was 23% lower than the maximum aerobic metabolism achieved during forced running on a treadmill (VO_2_run; A lines: 21%, C lines: 25%; Supplementary Materials in Jaromin et al., 2018). Interestingly, in subsequent generations VO_2_swim was still lower than VO_2_run in C lines, while in A lines the difference decreased and in generations 19–21 the trait values were practically the same (Jaromin et al., 2016, 2018). Thus, in our model system both the physiological abilities (the aerobic capacity) and the behavioral trait (willingness to perform at the upper level of abilities) evolved.

It is well known that changes in neuronal signaling pathways can lead to an altered response to drugs (Rhodes et al., 2005; Napolitano et al., 2010). For example, drugs that stimulate activity in healthy people, can depress it in patients with dopamine signaling impairment such as Attention Deficit Hyperactivity Disease (Jafarinia et al., 2012). Thus, pharmacological manipulations can be effectively applied to investigate the involvement of particular neurotransmitters and their receptors in exercise performance of both animals and humans (review in Roelands and Meeusen, 2010). It is especially useful when a large group of animals needs to be measured, as it is in the case of selective breeding experiments. The approach has been successfully used within the framework of other selection experiments (e.g., Rhodes et al., 2001; Rhodes and Garland, 2003; Keeney et al., 2012). For instance, the activity of mice selected for high wheel-running behavior decreased after administration of a dopamine reuptake inhibitor, i.e., a drug that effectively increases the dopaminergic signaling, but the drug had little effect on unselected, control mice (Rhodes et al., 2001). This differential drug response suggested differences in dopamine signaling between high wheel-runners and control mice. Indeed, the high-performance liquid chromatography analysis of brain tissue demonstrated a decreased level of dopamine in high wheel-runners (Waters et al., 2013).

We applied the pharmacological approach to investigate which neuronal signaling pathways underlie the differences between the A and C-line voles in the willingness to undertake intensive physical activity. In our previous research, a decrease in VO_2_swim was observed after injections of the reuptake inhibitors of dopamine (8%; vanoxerine), serotonin (6%; fluoxetine) or norepinephrine (8%; reboxetine), but the response differed between the A and C-line voles only in the case of reboxetine (Jaromin et al., 2018). Thus, the results corroborated the involvement of norepinephrinergic, serotoninergic and dopaminergic signaling in motivation to undertake the locomotor activity, but provided an evidence of a selection-related modification only for the norepinephrinergic signaling pathway. As the monoaminergic signaling is modulated by eCB (e.g., Tzavara et al., 2003; review in Kano et al., 2009), in this study we tested the hypotheses that eCB signaling pathway (1) affects the voles performance in the aerobic exercise trials, and (2) has been modified in the selection process. To this end, we asked (1) if administration of an eCB reuptake inhibitor (AM404) and a CB1 receptor antagonist (Rimonabant) affect the level of exercise metabolism, and (2) whether the effect differs between the A-selected and C-control lines of the voles.

## Materials and Methods

### Animals

This work is based on a typical scheme of artificial selection studies, in which lines of organisms are selected for many generations for high or low values of a particular trait, and then, after the trait is clearly differentiated, correlated responses in other traits are analyzed, and manipulative experiments are performed to investigate interactions between the genetically based effects of selection and environmental effects of the manipulation (Garland and Rose, 2009).We used bank voles (*Myodes glareolus*) from generation 22 of the artificial selection experiment in which four replicate lines of voles are selected for the 1-min-maximum rate of oxygen consumption achieved during 18 min swimming trial at 38°C (VO_2_swim; “A” – aerobic lines) and four replicate lines of unselected, randomly bred voles (“C” – control lines). Details of the selection experiment and animal maintenance are described in Sadowska et al. (2008, 2015). In generation 22, we measured VO_2_swim of 608 voles from A and 178 voles from C lines at the age of 73–85 days (selection trial). Voles that were not able to swim for 15 min or were diving were not chosen for the pharmacological experiment (about 8% of the A-line and 20% of the C-line voles were excluded for these reasons). In that way, we chose 6 females and 6 males from each of the A and C lines (a total of 96) for each of the two experiments (with AM404 and with Rimonabant). In each experiment each individual represented a different family.

All procedures associated with the breeding, selection and experimental procedures were approved by the 1st Local Ethical Committee on Animal Testing at the Jagiellonian University in Krakow, Poland (Decision No. 68/2012) and the 2nd Local Institutional Animal Care and Use Committee (IACUC) in Krakow, Poland (Decision No. 73/2016).

### Pharmacological Experiments

At the age of 78–90 days the voles underwent two measurements of oxygen consumption during swimming (VO_2_swim) and about 2 weeks later two measurements during forced running (VO_2_run) after pharmacological manipulation. According to literature, AM404 and Rimonabant in the doses range of 0.3–10 mg/kg effectively change physical activity in many different rodent models (e.g., Adamczyk et al., 2008; Keeney et al., 2008; Rasmussen and Hillman, 2011; Häring et al., 2013; Llorente-Berzal et al., 2015; Manna and Umathe, 2015). We chose the highest dosages of the drugs applied in other studies and we performed a pilot study to exclude the possibility that these dosages were too high for bank voles (e.g., causing immediate sedation, which would preclude swimming trials; unpublished data). In the proper experiment, with eCB reuptake inhibitor (AM404), the animals received an injection of AM404 (10 mg/kg) or pure vehicle, in which the drug was dissolved (DMSO:Tween80:saline 1:0.5:8.5). In the other experiment, with eCB antagonist (Rimonabant), the animals were injected with either the Rimonabant (10 mg/kg) or the same vehicle (the level of VO_2_swim after the vehicle injection did not differ from that after saline injection; unpublished results of pilot trials). The injections were administered in random order and we balanced the number of each kind of injection administered to voles from particular line in each trial (i.e., we used a randomized crossover, counterbalanced design). We maintained at least one week break between trials to avoid a carry-over effect. All injections were administered intraperitoneally, in a volume of 10 mL/kg, 30 min before the measurement. AM404 (Tocris, Abington, United Kingdom) and Rimonabant (Cayman Europe, Tallinn, Estonia) were diluted in a mixture of DMSO (Tocris, Abington, United Kingdom), Tween80 (Sigma-Aldrich, Saint-Louis, United States) and saline ca. 2 h before each trial.

### Respirometric Measurements

The respirometric measurements were performed with open-flow, positive pressure respirometric systems (scheme on Figure 10.2A in Lighton, 2008), similar to those used in our earlier experiments on bank voles (Sadowska et al., 2005, 2015; Jaromin et al., 2018). Fresh air taken from outside of the building was dried with silica gel and pumped through the respirometric chamber at about 2,000 ml/min (STPD, measured before the chamber). The air flow was controlled with mass-flow controllers (GFC17 or GFC171S, Aalborg, Orangeburg, NY, United States) and the exact flow values were determined with high precision glass rotameters (ROTA model L2.5/100). A sample of excurrent air (about 200 ml/min) was dried with ND2 gas sample drier (Sable Systems, Las Vegas, NV, United States) and with chemical absorber (magnesium perchlorate), and directed to FC10 or FC-10a oxygen and CA-2A carbon dioxide analyzers (Sable system, Las Vegas, NV, United States). We performed calibration of the gas analyzers according to FC10, FC10a and CA-2A manuals. The oxygen analyzers were calibrated at one-point against clean, dry air, assuming it has a concentration of 20.95% oxygen. In case of the carbon dioxide analyzer, the adjustment of zero was performed with respect to carbon dioxide-free and dry air (carbon dioxide was removed with Ascarite), and the adjustment of span with a reference calibration gas containing 0.8% CO_2_. The analyzers recorded the gas concentrations at 1-s intervals. The rates of oxygen consumption were calculated with appropriate respirometric equations (Supplementary Materials in Sadowska et al., 2015) and corrected for effective volume to achieve instantaneous rates (Bartholomew et al., 1981; Lighton, 2008).

To measure the VO_2_swim we used simultaneously two systems with respirometric chambers (15 cm diameter 3-liter glass jars) partly filled with water (38°C). After recording the initial baseline for 30 s, the chamber was opened and the animal was delicately placed on the water surface and allowed to freely swim. Preliminary observations indicated that oxygen consumption is higher when the vole swims without any additional load, presumably because with the additional load they had difficulty with keeping the head above surface and hence with breathing. Thus, we did not applied any additional load. The swimming trial lasted 18 min, except for 7 cases, when the animals were getting weak (the VO_2_swim was decreasing) and we stopped the test. However, these animals achieved their VO_2_swim at least 80 s before the test was stopped, and therefore the decision to stop the trials at a particular moment did not influence the VO_2_swim value. After removing the animal from the chamber, the chamber was closed, and after sufficient washout time (about 2 min) a 30-s final baseline was recorded.

The 1-min-maximum forced-running rate of oxygen consumption (VO_2_run) was measured in a respirometric treadmill for rodents (BTU-100-10-M, Bio-Sys-Tech, Bialystok, Poland). The animals were forced to run by mild electric shock (0.5 mA) generated by bars located at the end of the moving belt. The fur of the abdomen and hind legs was moistened with warm water (38°C) that contained a drop of dog shampoo, to increase electric conductivity. Without that procedure the animals ignored the mild electric shocks. To decrease velocity at which the voles achieved the maximum effort (typically 25–75 m/min), and hence make the procedure safer to the animals, the treadmill was inclined by 10°. We also placed two ping-pong balls at the end of the moving belt, which helped the animals learn faster to avoid the electric bars. After recording the initial baseline for 30 s, the vole was placed in the chamber and the chamber was closed. The treadmill started to move at 5 m/min one minute after starting the trial and the speed was increased by 5 m/min every minute. The test lasted till exhaustion, i.e., till the animal was unable to keep pace with the moving belt (typically 5–15 min). The trial was stopped when the vole was pushed the third time onto the electric bars. As the vast majority of the voles achieved VO_2_run well before the trial termination, the decision of observer about the exact moment of ending the trial had no practical effect on the results. After removing the vole from the chamber, the chamber was closed, and after sufficient washout time (about 2 min) a 30-s final baseline was recorded. The measurements were preceded by two habituation trials to familiarize the animals with the treadmill.

### Statistical Analysis

We applied nested analysis of covariance (ANCOVA) mixed models implemented in SAS 9.3 (SAS Institute Inc., Cary, NC, United States) Mixed procedure (SAS Institute Inc., 2011), with REML estimation method and variance components constrained to non-negative values. As several complex models were fitted, in addition to the description presented here, we provide a commented SAS code used for the analyses (Supplementary Tables S1–S3). All the models included main fixed factors of line Type (selected vs. control), Sex, and line Type × Sex interaction, and random effects of replicate Line (nested in line Type) and Sex × Line interaction. The models included also a fixed cofactor (consecutive Litter Number of a given female, in which the individual was born: 1, 2 or 3) and two covariates: Age, and Litter Size. The models in which the VO_2_swim or VO_2_run was the dependent variable included also Body Mass as a covariate. The models concerning VO_2_swim included also Respirometric system number as a cofactor. This basic model structure was further expanded to accommodate additional factors adequate for particular datasets:

(1) The models used for analyzing the selection-trial body mass and VO_2_swim, measured in all voles from generation 22, included random effect of Family nested in Line. This effect was introduced to properly handle non-independence of observations obtained on individuals from the same full-sib families.
(2) To compare the 1-min maximum rates of oxygen consumption achieved by the same individuals during swimming and running (VO_2_swim vs. VO_2_run), the basic model structure was expanded to a repeated-measures design (with individuals as subjects). The analysis was performed for results obtained only in trials without application of the drugs (i.e., only after the vehicle injections), but pooled from the two pharmacological experiments. The model included within-subject fixed factors of Exercise type (swim vs. run; a repeated measure factor) and Exercise type × line Type interaction, and random effect of replicate Line × Exercise type. Because the data pooled from two experiments included observations on siblings, the model included also between-subject random effect of Family nested in Line.
(3) Because the pharmacological manipulation was applied at the within-individual level, a repeated measures model was also used to analyze data from the pharmacological experiments. First, we tested the additive effect of the drugs on 1-min-maximum swim-induced VO_2_ (VO_2_swim), the time of achieving VO_2_swim, whole swimming trial mean VO_2_ (mean VO_2_swim), and 1-min-maximum run-induced VO_2_ (VO_2_run), separately for the two experiments (with AM404 or Rimonabant). These models included the following within-subject (within-individual) effects: fixed factor of Drug (depending on the experiment: AM404 vs. vehicle or Rimonabant vs. vehicle), Drug × line Type interaction, trial Number as the repeated measure factor, and random replicate Line × Drug effect. The effect of Family was not included in these models, because each vole used within one experiment (AM404 or Rimonabant) represented a different family. Based on Akaike information criteria (AIC) we chose models with equal residual variances for the replicated trials as better than those with unequal variances.
(4) Next, we used the repeated measures models to test the multiplicative effect of the drugs. Here the dependent variable was the ratio of a VO_2_ achieved after drug to that after vehicle (proportional response). The analysis was performed for the VO_2_swim and VO_2_run ratios in one model, but separately for the two experiments (with AM404 or Rimonabant). These models included within-subject fixed factors of the Exercise type (swim vs. run, a repeated measure factor) and Exercise type × line Type interaction, and random effect of replicate Line × Exercise type.

All initial models included also interactions between Sex and main factors. However, these effects were not of main interest and were excluded from the final models when not significant (*p* > 0.05). To test for the homogeneity of slopes, initial models for VO_2_swim and VO_2_run included also interactions between Body Mass and the main factors, which were excluded from the final models when not significant. The slope for selection-trial VO_2_swim was significantly steeper in A than in C lines and the heterogeneous slopes had to be retained in the final model. In the analyses of data from the pharmacological experiments the differences of slopes were not statistically significant. However, these analyses were based on a dataset smaller than that available for selection trial, and, consequently, the power to detect the slope heterogeneity was lower. Moreover, in our earlier work we observed significant differences between the slopes in A and C lines (Jaromin et al., 2016, 2018; Rudolf et al., 2017). Therefore, we decided to retain the heterogeneous slopes also in the ANCOVAs performed on data from pharmacological experiments. Retention of the heterogeneous slopes in the models precluded a general test of difference between the level of VO_2_ in A and C lines. Therefore, to check whether the levels of VO_2_ differ between the line Types in the range of observed body mass, we tested this effect at specific values of the body mass covariate (option “at” in LSMEANS statement in the SAS Mixed procedure; SAS Institute Inc., 2011). For a more transparent presentation of the results, the adjusted Least-Squares Means (LSM) presented on Figures were calculated at mean values of covariates (body mass, age and Litter size: 24 g, 78 days and 6 in selection trial, and 24 g, 85 days and 5.5 in further trials).

We checked distribution of residuals by inspecting SAS-generated diagnostic graphs (plots of residual vs. predicted values, histograms, and quantile plots). We excluded statistical outliers from the analysis when the studentized residual was higher than 3.0. In that way we excluded one outlier from the analysis of proportional response to AM404 and two outliers from the analysis of proportional response to Rimonabant. In the analysis of selection-trial VO_2_swim for all voles in generation 22, i.e., a much larger sample, we increased the threshold to 3.5 (two outliers were excluded). The exclusion of the outliers improved the distribution of residuals, but did not influence final conclusions. The residuals distribution in the analysis of the time of achieving VO_2_swim was somewhat heteroscedastic. Square-transformation improved the residuals distribution, but results of the analysis were similar and lead to the same conclusions. Therefore, for the clarity of presentation, we report here the raw data analysis. In all models, we used the Satterthwaite’s approximation to calculate the effective denominator degrees of freedom (dfs) of the F statistics or df in t statistics (SAS Institute Inc., 2011). Shortly, in the model with nested random effects, the appropriate denominator dfs are computed as combination of the dfs of appropriate random effects (e.g., the nested factor effects and residual term) weighted by variance contributed by the effects. When the variance of the nested factor (in our case replicate Lines nested in line Type) approaches zero, the appropriate denominator df approach the dfs for residual error term (which reflects the fact that in the absence of the among-group variance, individual observations can be effectively treated as statistically independent). Thus, when the Satterthwaite method is used, the denominator dfs can take any real value from the range between the df of the nested random factor and the df of residual term (but for clarity of presentation, the Satterthwaite dfs were rounded to whole numbers).

Unless otherwise stated, we used Tukey-Kramer for multiple pairwise comparisons between groups. We tested the significance of random factors with likelihood ratio test (LRT), using models such as described above, but with the variance constrain relaxed (“nobound” option in SAS Mixed procedure).

In the Results section, we report main effects of line Type and Drug. Comprehensive statistical results are presented in Supplementary Materials: descriptive statistics (Supplementary Table S4), detailed information about all others fixed factor effects (Supplementary Tables S4–S9), and estimation of variance components and LRT tests (Supplementary Table S10).

## Results

**FIGURE 1:**
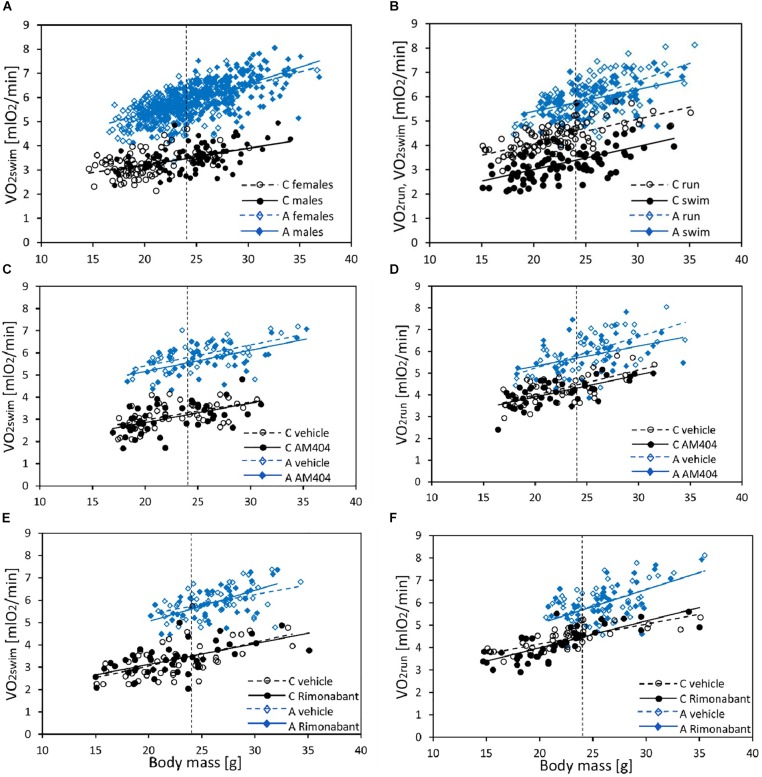
The relationship between swim-induced (VO_2_swim) or run-induced (VO_2_run) 1-min maximum rate of oxygen consumption and body mass in bank voles, and the effects of selection, sex, and pharmacological manipulation. **(A)** VO_2_swim in all individuals from generation 22 (C – Control lines, A – Aerobic lines) tested as a part of the selection experiment (selection-trial results). **(B)** The effect of Exercise Type (VO_2_swim vs. VO_2_run) in voles from C (*N* = 96) and A (*N* = 96) lines (voles used in AM404 and Rimonabant experiments combined). **(C,D)** The effect of AM404 [an endocannabinoid (eCB) reuptake inhibitor] on VO_2_swim (C) and VO_2_run (D): repeated trials in a subsample of voles from C (*N* = 48) and A (*N* = 48) lines. **(E,F)** The effect of Rimonabant (a cannabinoid receptor CB1 antagonist) on VO_2_swim **(E)** and VO_2_run **(F)**: repeated trials in a subsample of voles from C (*N* = 48) and A (*N* = 48) lines. Dotted lines indicate the mean body mass for which the adjusted least-squares means were calculated (shown in Figure 2–4 and Supplementary Table S4).

In the selection trial, body mass ranged from 14.6 to 36.9 g and mean body mass (24 g) did not differ significantly between A and C lines (*F*_1,6_ = 1.93, *p* = 0.21, Figure 1A and Supplementary Table S4). Males were heavier than females (*F*_1,7_ = 102, *p* < 0.0001) and the line Type × Sex interaction was not significant (*F*_1,7_ = 1.09, *p* = 0.33). The 1 min-maximum swim-induced (VO_2_swim) oxygen consumption increased with Body Mass and the slope of the relationship between VO_2_swim and Body Mass was steeper in A-lines (slope difference ± SE: 0.05 ± 0.015, *t*_191_ = 3.58, *p* = 0.0004; Figure 1A and Supplementary Table S5). At the minimum body mass in the dataset (14.6 g), voles from A lines achieved a higher VO_2_swim than voles from C lines (*t*_86_ = 11.5, *p* < 0.0001) and, as the difference increased with body mass, we can conclude that the difference in VO_2_ between voles from A and C lines was significant for the whole range of body mass (at mean body mass = 24 g, *t*_8_ = 18.9, *p* < 0.0001). The mean values of VO_2_swim in two subsets of A- and C-line voles sampled for the pharmacological experiments were almost exactly the same as the respective means for all the animals used in the selection trial (Supplementary Table S4).

**FIGURE 2:**
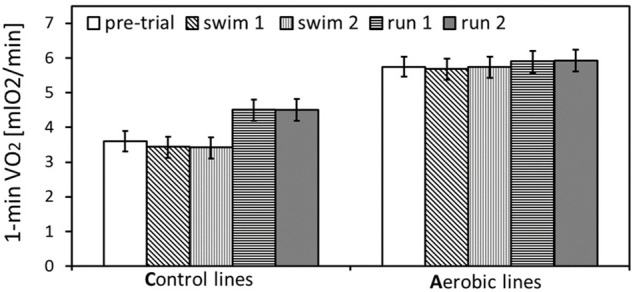
The maximum rate of oxygen consumption achieved during selection trial and pharmacological trials: swim trials 1 and 2, and run trials 1 and 2 (only the trials after placebo-vehicle injections), in bank voles from selected A lines (*n* = 96) and unselected C lines (*n* = 96). The adjusted least squares means for the mean body mass (24 g) with 95% confidence limits are presented [LSM(95%CL) note: an overlap of the confidence limits does not indicate a lack of difference between repeated measurements at different conditions, because the confidence limits are based on among-individual variation at particular conditions, whereas the inferences concerning differences between conditions are based on within-individual comparisons. See Table 1 for results of the proper significance tests].

**FIGURE 3:**
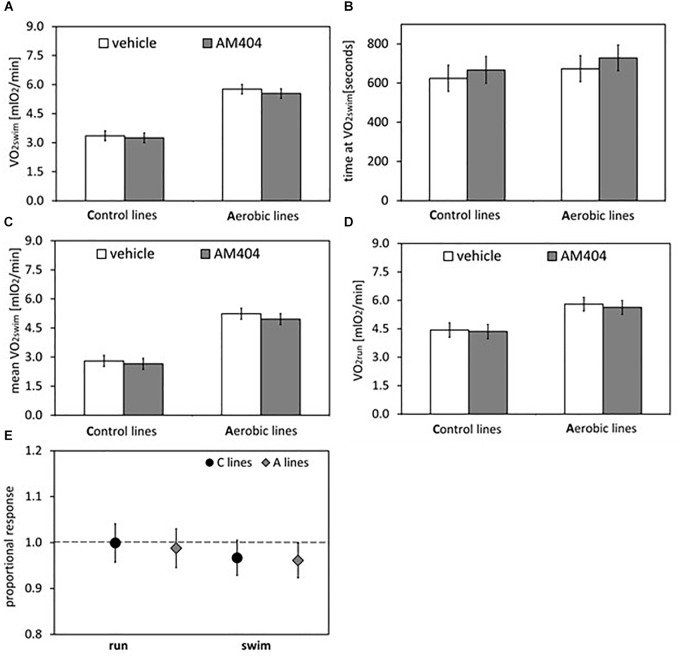
Summary of the main results from experiment with AM404 – an endocannabinoid reuptake inhibitor: repeated trials in a subsample of voles from selected A (*n* = 48) and unselected C lines (*n* = 48). **(A)** 1-min maximum swim-induced oxygen consumption (VO_2_swim). **(B)** The time when voles achieved the VO_2_swim. **(C)** Whole-trial mean VO_2_swim (typically about 15 min). **(D)** 1-min maximum run-induced oxygen consumption (VO_2_run). **(E)** The ratio of VO_2_swim or VO_2_run achieved after drug to the one after vehicle (proportional response). Bars represent adjusted least squares mean with 95% confidence limits (LSM[95%CL] Note: as explained in Figure 2 legend, an overlap of the confidence limits does not indicate that a difference is not significant. See Figure 5 and Tables 1, 2 for results of the proper significance tests).

**FIGURE 4:**
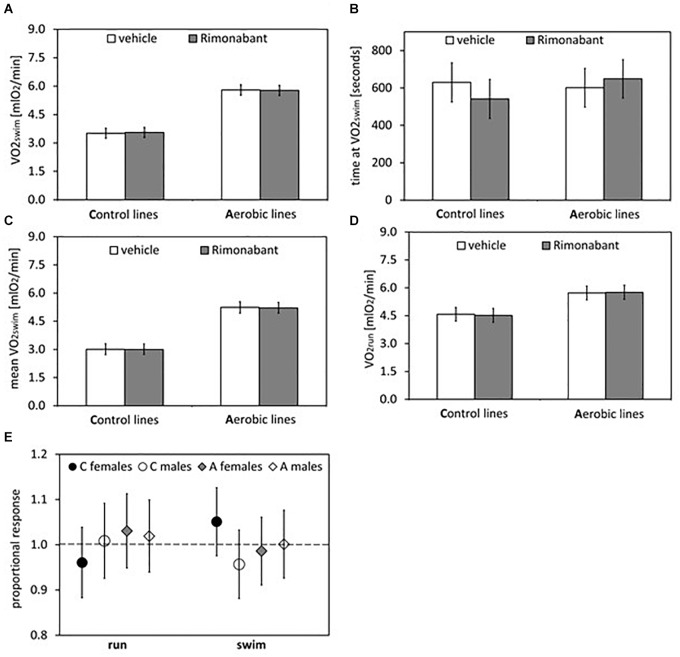
Summary of the main results from experiment with Rimonabant – cannabinoid receptor CB1 antagonist: repeated trials in a subsample of voles from selected A (*n* = 48) and unselected C lines (*n* = 48). **(A)** 1-min maximum swim-induced oxygen consumption (VO_2_swim). **(B)** The time when voles achieved the VO_2_swim. **(C)** Whole-trial mean VO_2_swim (typically about 15 min). **(D)** 1-min maximum run-induced oxygen consumption (VO_2_run). **(E)** The ratio of VO_2_swim or VO_2_run achieved after drug to the one after vehicle (proportional response). Bars represent adjusted least squares mean with 95% confidence limits [LSM(95%CL) Note: as explained in Figure 2 legend, an overlap of the confidence limits does not indicate that a difference is not significant. See Figure 5 and Tables 1, 2 for results of the proper significance tests].

In the analysis concerning the exercise type effect (combined data from both pharmacological experiments), body mass ranged from 14.7 to 35.5 g and mean body mass (24 g) did not differ between A and C lines (*F*_1,6_ = 5.55, *p* = 0.06, Figure 1B and Supplementary Table S4). Males were heavier than females (*F*_1,6_ = 116, *p* < 0.0001) and the line Type × Sex interaction was not significant (*F*_1,6_ = 0.0, *p* = 0.97). Both the 1-min-maximum swim-induced (VO_2_swim) and run-induced (VO_2_run) oxygen consumption increased with Body Mass (Figure 1B and Supplementary Table S5). Although the line Type × Body Mass interaction was not significant in this analysis, we decided to retain it in the final models (see Methods). Overall ANCOVA for mean body mass (24 g) revealed that both the selection, the exercise type, and their interaction affected the rate of metabolism (Type: *t*_6_ = 8.65, *p* = 0.0001; Exercise Type: *F*_1,7_ = 67.1, *p* < 0.0001, line Type × Exercise Type: *F*_1,7_ = 26.9, *p* = 0.0011; Figure 1B, 2, 3A,D, 4A,D and Table 1). Voles from A lines achieved a generally higher VO_2_ than voles from C lines (partitioned analysis; VO_2_swim: *F*_1,8_ = 102.2, *p* < 0.0001; VO_2_run: *F*_1,8_ = 35.6, *p* = 0.0003). Voles from the A lines had also a higher “mean VO_2_swim,” i.e., the average VO_2_ in the entire swimming trial (usually about 15 mins; Figure 3C, 4C and Table 1). The analyses performed for the minimal and maximal body mass showed the same pattern for the differences between A and C lines in all the aerobic performance traits, thus we could conclude that the same conclusions concern the whole range of body mass. In A lines VO_2_run was only 3% higher than VO_2_swim and the difference was not significant (*F*_1,7_ = 4.48, *p* = 0.07), while in C lines VO_2_run was 32% higher than VO_2_swim (*F*_1,7_ = 89.8, *p* < 0.0001).

**Table 1:** Main results of mixed ANCOVA models (effects of selection, pharmacological treatment and their interaction) for the data obtained in the two experiments with pharmacological manipulation (AM404 – an endocannabinoid reuptake inhibitor or Rimonabant – cannabinoid receptor CB1 antagonist).

Experiment	Line type		Drug		Line type × Drug	
Trait	t (df)	*P*-value	F (Ndf, Ddf)	*P*-value	F (Ndf, Ddf)	*P*-value
**AM404 experiment**						
VO_2_swim	15.6 (8)	<0.0001	7.82 (1,6)	0.03	1.00 (1,6)	0.35
time at VO_2_swim	1.45 (88)	0.15	2.61 (1,94)	0.11	0.04 (1,94)	0.84
mean VO_2_swim	13.6 (8)	<0.0001	15.4 (1,6)	0.007	1.32 (1,6)	0.29
VO_2_run	5.90 (7)	0.0005	3.39 (1,79)	0.07	0.47 (1,79)	0.49
**Rimonabant experiment**						
VO_2_swim	14.2 (21)	<0.0001	0.02 (1,8)	0.90	0.12 (1,8)	0.73
time at VO_2_swim	0.69 (6)	0.52	0.43 (1,92)	0.52	4.64 (1,92)	0.03
mean VO_2_swim	12.6 (9)	<0.0001	0.08 (1,7)	0.78	0.02 (1,7)	0.91
VO_2_run	5.76 (7)	0.0006	0.01 (1,6)	0.94	0.15 (1,6)	0.71

*VO_2_swim, VO_2_run – 1-min maximum swim-or run-induced rate of oxygen consumption; mean VO_2_swim, whole-trial mean VO_2_swim; F or t statistics; df, degrees of freedom; Ndf, numerator df; Ddf, denominator df; see Supplementary Tables S7, S8 for results concerning additional factors in the ANCOVA models.*

**FIGURE 5:**
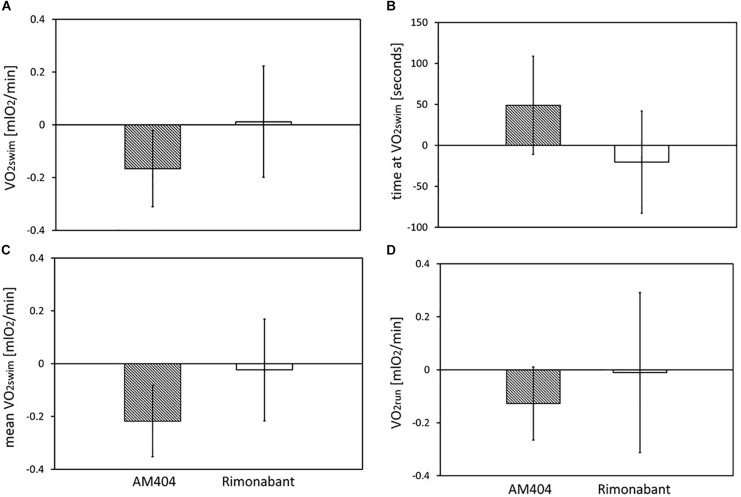
The effects of Rimonabant (cannabinoid receptor CB1 antagonist) and AM404 (endocannabinoid reuptake inhibitor on the measured traits): **(A–D)** Mean within-individual differences between the values obtained after drug treatment and vehicle injection [**(A)** 1-min maximum swim-induced oxygen consumption (VO_2_swim); **(B)** the time when voles achieved the VO_2_swim; **(C)** whole-trial mean VO_2_swim; **(D)**1-min maximum run-induced oxygen consumption (VO_2_run)]. Bars represent the least squares estimates of the mean differences with 95% confidence limits.

The administration of eCBs reuptake inhibitor (AM404), resulted in decreased VO_2_swim (4%, *F*_1,6_ = 7.71, *p* = 0.03), mean VO_2_swim (5%, *F*_1,6_ = 15.4, *p* = 0.007) and tended to decrease VO_2_run (2%, *F*_1,79_ = 3.39, *p* = 0.07), but the line Type × Drug interaction had no significant effect on any of the three traits (*p* ≥ 0.29; Figure 1C,D, 3A,C,D, Table 1 and Supplementary Table S4). The time of achieving VO_2_swim did not differ significantly between A and C lines (*t*_88_ = 1.45, *p* = 0.15) and was not affected by the Drug (*F*_1,94_ = 2.61, *p* = 0.11) or line Type × Drug interaction (*F*_1,94_ = 0.04, *p* = 0.84; Figure 3B and Table 1). Note that in the additive models (results reported above) the line Type × Drug interactions conveys the information on the hypothetical effect of selection on eCB pathways. On the other hand, in the analysis of the proportional response to the drug (the ratio of VO_2_ achieved after drug to that after vehicle) the hypothetical effect of selection on eCB pathways is expressed directly in the line Type effect. The proportional response, however, was not influenced significantly by the line Type (*t*_12_ = 0.45, *p* = 0.66, Figure 3E and Table 2), Exercise type (*F*_1,13_ = 2.71, *p* = 0.12) or line Type × Exercise type interaction (*F*_1,13_ = 0.02, *p* = 0.88).

**Table 2:** Proportional response – the ratio of VO_2_ achieved after drug to the one after vehicle in voles used in the two experiments with pharmacological manipulation (AM404 – an endocannabinoid reuptake inhibitor or Rimonabant – cannabinoid receptor CB1 antagonist).

Experiment	Line type		Exercise type		Line type × Exercise type	
	t (df)	*P*-value	F (Ndf, Ddf)	*P*-value	F (Ndf, Ddf)	*P*-value
AM404 experiment	0.45 (12)	0.66	2.71 (1,13)	0.12	0.02 (1,13)	0.88
Rimonabant experiment^∗^	0.34 (6)	0.75	0.15 (1,5)	0.72	2.10 (1,6)	0.20

*Main results of mixed ANCOVA models (effects of selection, exercise type and their interaction) are presented; F or t statistics; df, degrees of freedom; Ndf, numerator df; Ddf, denominator df; see Supplementary Table S7, S8 for results concerning additional factors in the ANCOVA model. ^∗^Sex × Exercise type: F_1,80_ = 3.39, p = 0.07; Line type × Sex × Exercise type interaction: F_1,81_ = 7.15, p = 0.009.*

The administration of eCB receptor antagonist (Rimonabant) had no effect on VO_2_swim, mean VO_2_swim or VO_2_run, and the line Type × Drug interaction was not significant, either (*p* ≥ 0.70; Figure 1E,F, 4A,C,D, Table 1 and Supplementary Table S4). The time of achieving VO_2_swim did not differ between the A and C lines (*t*_6_ = 0.69, *p* = 0.52) or Drug (*F*_1,92_ = 0.4, *p* = 0.52), but the line Type × Drug interaction was significant (*F*_1,92_ = 4.64, *p* = 0.03; Figure 4B and Table 1). This is because injection of the drug tended to decrease the time of achieving VO_2_swim in C lines, but increase it in A lines. However, in the analyses performed for A and C lines separately, the drug effect was not significant (in A lines: *F*_1,46_ = 1.80, *p* = 0.19, C lines: *F*_1,45_ = 2.86, *p* = 0.10). In the analysis of proportional response (VO_2_ achieved after drug to that after vehicle) the effects of line Type (*t*_6_ = 0.34, *p* = 0.75), Exercise type (*F*_1,5_ = 0.15, *p* = 0.72), line Type × Exercise type (*F*_1,6_ = 2.10, *p* = 0.20) or Sex × Exercise type interaction (*F*_1,80_ = 3.39, *p* = 0.07) were not significant (Figure 4E and Table 2). However, Line Type × Sex × Exercise type interaction was significant (*F*_1,81_ = 7.15, *p* = 0.009). That was because the proportional response was higher in females than in males from C lines during swimming, but during running the opposite was observed (the response was lower in females than in males; Tukey comparisons: swim C-lines: females vs. males: *t*_123_ = 3.28, *p* = 0.03, Supplementary Table S9).

## Discussion

As expected, in generation 22 bank voles from the A-selected lines achieved 65% higher mass-adjusted swim-induced aerobic metabolism (VO_2_swim) than voles from C-control lines (Figure 1, 2). Similarly as in the previous generations (Jaromin et al., 2016, 2018), in C lines the VO_2_swim was 24% lower than the maximum forced-running aerobic metabolism (VO_2_run), but in the A lines the levels of VO_2_swim and VO_2_run were nearly the same (Figure 1B, 2). Thus, the voles from A lines evolved both the increased aerobic capacity and willingness to voluntarily undertake physical activity at the level approaching their physiological limit. To investigate the neuronal mechanism underlying the differences in motivation to be active between voles from A and C lines we measured VO_2_swim and VO_2_run after injections of drugs modulating eCB signaling.

Application of the CB1 receptor antagonist (Rimonabant) had no significant effect on VO_2_swim or VO_2_run (Figure 4A,D), but it caused a differential effect on the time of achieving VO_2_swim (Figure 4B). Specifically, voles from C lines achieved VO_2_swim faster, while those from A lines later after Rimonabant injection when compared to vehicle (significant line Type × Drug interaction). However, the drug effects in the analyses performed for A and C lines separately were not significant. Interestingly, the proportional response to Rimonabant during swimming (VO_2_swim after Rimonabant to that after vehicle) was higher in females than males from C lines and during running it was the opposite (Figure 4E). The sex differences in the response to eCB-modulating drugs were reported in literature, e.g., eCB agonist (tetrahydrocannabinol) increased locomotor activity in females, but had no effect in males (Wiley, 2003). In another selection experiment, Rimonabant decreased running to a greater degree in female mice selected for high voluntary wheel running than in selected males or control, unselected mice (Keeney et al., 2008). Current literature provides strong evidence of sex differences in physical activity level in humans and rodents that can be of genetic, hormonal or neuronal basis, however the exact mechanism remains unclear (review in Rosenfeld, 2017). To summarize, the effects of Rimonabant on the traits measured in bank voles are weak, and the results are not conclusive. However, the marginally significant interactions indicate that further investigation of the effects of other eCB antagonists is worthwhile.

On the other hand, administration of the eCB reuptake inhibitor (AM404) significantly decreased the 1-min-maximum VO_2_swim (4%; Figure 3A), mean VO_2_swim (5%; Figure 3C), and it tended to decrease VO_2_run in all voles (2%; Figure 3D). The literature provides confusing results concerning the influence of eCB-modulating drugs on the voluntary physical activity. Although direct brain microinjections of eCB agonist increased locomotor activity in rats and established conditioned place preference (Zangen et al., 2006), the systemic administration of eCB agonists either increased or decreased locomotor activity (review in Tantimonaco et al., 2014). According to Almeida et al. (2014), open-field locomotor activity increased in wild-type rats and decreased in hyperactive rats after systemic injection of AM404. However, in other studies AM404 decreased the open-field locomotor activity also in wild-type rats (Beltramo et al., 2000) or had no significant effect (Adamczyk et al., 2008). Moreover, both the agonist and antagonist of CB1 receptor decreased voluntary wheel running in mice (Keeney et al., 2008, 2012). It seems that the results of different tests designed to measure voluntary activity (e.g., open filed, wheel running, home-cage activity) may not be correlated (Careau et al., 2012). The contradictory findings can result not only from different conditions of the experiment, but also from the complexity of the eCB system and the eCB-exercise interplay, which are still not fully recognized (Tantimonaco et al., 2014). As the response to drugs differs greatly between species and even strains (e.g., Petit-Demouliere et al., 2005), the decrease in voluntary physical activity after AM404 administration can be specific for bank voles.

The forced-running protocols are widely applied to measure the maximum capacity for physical activity, however it is also known that they do not warrant that the result is free from the behavioral responses, such as willingness to undertake the exercise (Smirmaul et al., 2013). What is more, the mild electric shocks, applied as stimulator in the VO_2_run tests, can cause an eCBs release (Guindon and Hohmann, 2009). Administration of AM404 decreased VO_2_run in the voles, but the effect was two times smaller than in case of VO_2_swim. It could be hypothesized that the application of AM404 affected not the motivation to undertake activity, but a regulatory or metabolic pathway determining the aerobic exercise capacity of the voles (even though, to our knowledge, such effects have not been reported). However, if this were the case, the application of AM404 would lead to a larger change of the forced-exercise VO_2_run than of the voluntary-exercise VO_2_swim. As the opposite was observed, we conclude that AM404 affected a mechanism regulating a behavioral aspect of the locomotor activity rather than the ability to perform work. Thus, the results are consistent with the hypothesis of involvement of the eCB signaling in mechanisms regulating motivation to undertake the activity. However, we cannot exclude a possibility that the AM404 affected some other behavioral aspect of activity, such as arousal, rather than motivation *per se*. Thus, the conclusion requires further support.

To investigate the effect of selection on eCB signaling pathway we tested line Type × Drug interactions in additive models and line Type effect in the proportional response analysis. Despite the complexity of eCB signaling, a distinct reaction to drugs administration in the A and C lines would indicate that eCB signaling pathway has been changed in the course of selection. However, although AM404 resulted in decreased aerobic metabolic rates both in the swimming and running trials, we did not find any differences in the response to AM404 between voles from A and C lines. This negative result does not exclude the possibility that eCB signaling has changed under the influence of selection, but with such a simple scheme of pharmacological manipulation some subtle modifications in the eCB signaling could remain undetected.

To summarize, the results provided further evidence for the involvement of eCB in the regulation of physical activity and especially in the willingness to undertake intensive exercise. However, the results did not support the hypothesis that an alternation of eCB signaling pathway have played a role in the evolution of increased aerobic exercise performance in our experimental evolution model. Because the pharmacological approach has several limitations (e.g., not complete specificity of the drugs and their systemic application), the conclusions require an additional support. As the selection experiment is continued, further studies can answer the question which neurobiological mechanism underlies the evolutionary changes in motivation to be active.

## Ethics Statement

All procedures associated with the breeding, selection and experimental procedures were approved by the 1st Local Ethical Committee on Animal Testing at the Jagiellonian University in Krakow, Poland (Decision No. 68/2012) and the 2nd Local Institutional Animal Care and Use Committee (IACUC) in Krakow, Poland (Decision No. 73/2016).

## Author Contributions

EJ, PK, and ES contributed to conception and design of the study. EJ organized the database and performed the statistical analysis. EJ wrote the first draft and sections of the manuscript. All authors contributed to manuscript revision, read, and approved the submitted version.

## Conflict of Interest Statement

The authors declare that the research was conducted in the absence of any commercial or financial relationships that could be construed as a potential conflict of interest.

## Abbreviations

2-AG: 2-arachidonoylglycerol
AEA: N-arachidonylethanolamine
eCB: endocannabinoid
VO2: rate of oxygen consumption
VO2run: the 1-min maximum rate of oxygen consumption achieved during running
VO2swim: the 1-min maximum rate of oxygen consumption achieved during swimming.
