# Supplementary material for: Is Experimental Evolution of an Increased Aerobic Exercise Performance in Bank Voles Mediated by Endocannabinoid Signaling Pathway?
Source: Front Physiol. 2019 May 28;10:640. doi: 10.3389/fphys.2019.00640 (PMC6546880; doi:10.3389/fphys.2019.00640)
Supplement: Supplementary file 1 [file Table_1.DOCX]

**Is experimental evolution of an aerobic exercise performance in bank voles mediated by endocannabinoid signaling pathway?**

Ewa Jaromin^1^, Edyta T. Sadowska, Paweł Koteja

Institute of Environmetal Sciences, Jagiellonian University, Krakow, Poland

^1^corresponding author: [jaromin.eva@gmail.com](mailto:jaromin.eva@gmail.com)

**SUPPLEMENTARY MATERIALS – FIGURES AND TABLES**

**
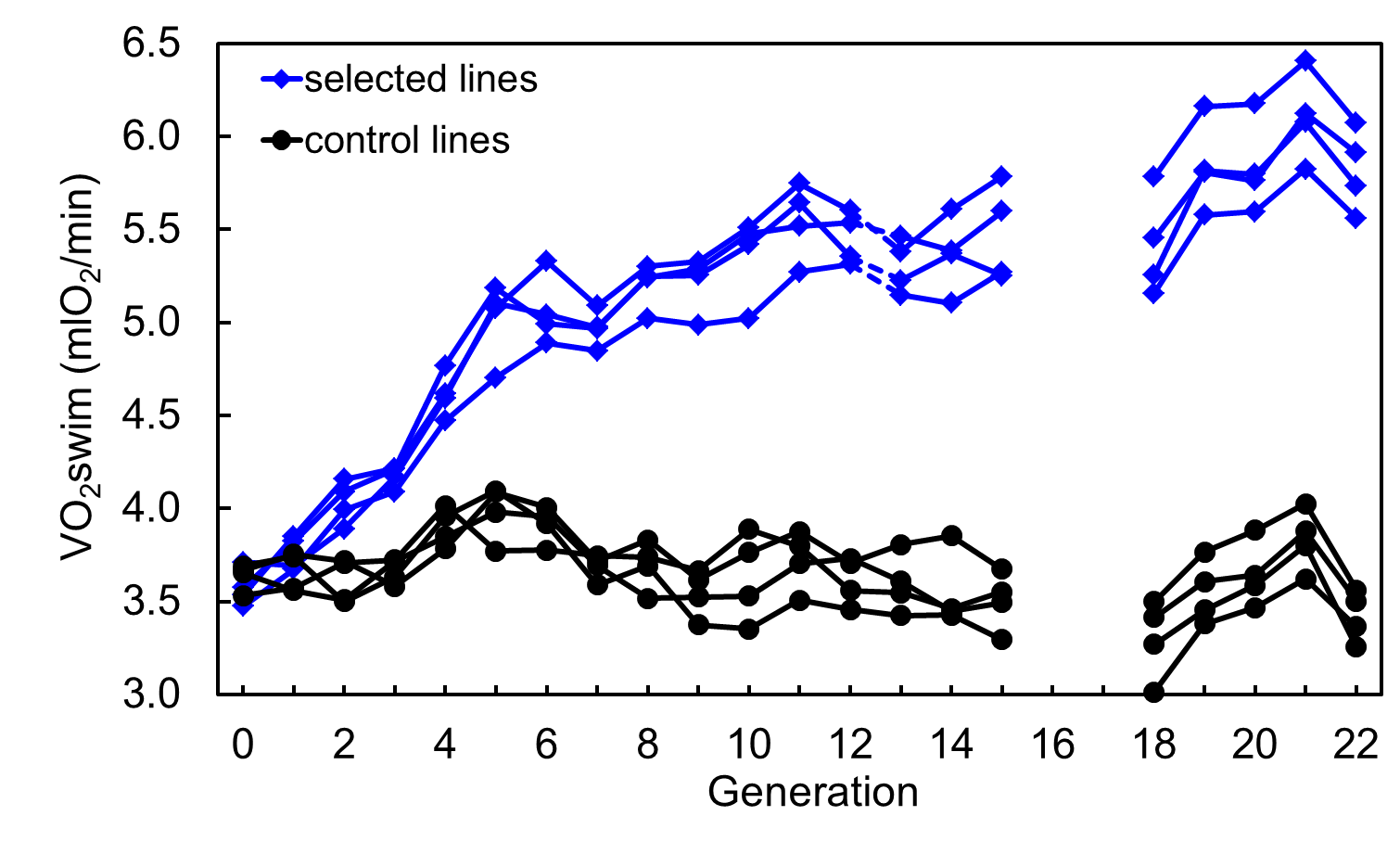
**

**Figure S1 The direct effect of selection for high 1-min maximum swim-induced rate of oxygen consumption (VO_2_swim) in bank vole** (replicate line means, not adjusted for body mass; in generations 12, 16 and 17 the selection was relaxed)

**Table S1 SAS statistical software codes.** To analyze the data we used nested analysis of covariance (ANCOVA) mixed models implemented in SAS 9.3 (SAS Institute Inc., Cary, NC, USA) Mixed procedure (with REML estimation method and variance components constrained to non-negative). The SAS Software codes for saturated and reduced models are presented (see Statistical analysis section in Methods in the main text). The description of variables and commends used in the codes are provided in the Tables S2 and S3.

| Model Type | Codes |
| --- | --- |
| The models used for analyzing the selection-trial VO_2_swim. Similar models, but without body mass as a covariate, were used to analyze selection-trial body mass. | |
| Saturated | **PROC** **Mixed** data=data method=reml alpha=**0.05** cl covtest ;  CLASS T S ID L Fam SysNu LitNu ;  MODEL Y = T S T*S BM T*BM S*BM T*S*BM SysNu LitNu LitS Age Date / ddfm=satterthwait ;  RANDOM L(T) Fam(L(T)) S*L(T) BM*L(T) BM*S*L(T);  **RUN** |
| Reduced | **PROC** **Mixed** data=data method=reml alpha=**0.05** cl covtest ;  CLASS T S ID L Fam SysNu LitNu ;  MODEL Y = T S T*S BM T*BM SysNu LitNu LitS Age / ddfm=satterthwait cl ;  RANDOM L(T) Fam(L(T)) S*L(T) BM*L(T) ;  LSMEANS T / cl at BM=**14.6 diff** ;  LSMEANS T / cl at BM=**24.0** **diff** ;  LSMEANS T*S / cl at means adjust=tukey ;  LSMEANS SysNu LitNu / cl at means;  **RUN** |
| The models used to compare VO2swim (after vehicle injection only) with VO2run. | |
| Saturated | **PROC** **Mixed** data=data method=reml alpha=**0.05** cl covtest ;  CLASS T S ExType ID L Fam LitNu Exp;  MODEL Y = T S ExType T*S T*ExType S*ExType T*S*ExType BM T*BM S*BM ExType*BM T*S*BM ExType*T*BM ExType*S*BM ExType*T*S*BM LitNu Exp LitS Age_t1 / ddfm=satterthwaite ;  RANDOM L(T) Fam(L(T)) S*L(T) ExType*L(T) ExType*S*L(T) BM*L(T) BM*S*L(T) ExType*BM*L(T) ExType*BM*S*L(T) ;  REPEATED ExType / type=cs subject=ID ;  **RUN** |
| Reduced | **PROC** **Mixed** data=data method=reml alpha=**0.05** cl covtest ;  CLASS T S ExType ID L Fam LitNu Exp;  MODEL Y = T S ExType T*S T*ExType BM T*BM LitNu Exp LitS Age_t1 / cl solution ddfm=satterthwaite ;  RANDOM L(T) Fam(L(T)) S*L(T) ExType*L(T) BM*L(T) ;  REPEATED ExType / type=cs subject=ID ;  LSMEANS T / cl at Age_t1=**85** at LitS=**5.5** at BM=**14.6** diff ;  LSMEANS T / cl at Age_t1=**85** at LitS=**5.5** at BM=**24.0** diff ;  LSMEANS S ExType T*S LitNu / cl at Age_t1=**85** at LitS=**5.5** at BM=**24.0** ;  LSMEANS ExType*T / adjust=tukey cl at Age_t1=**85** at LitS=**5.5** at BM=**24.0** ;  Slice T*ExType / cl at LSWN=**5.5** at age_p1=**85** at BM_p=**24.0**;  **RUN** |
| The models used for analyzing data from the two subsequent pharmacological trials. The presented models were used to analyze 1-min-maximum swim-induced VO_2_ (VO_2_swim), whole trial mean VO_2_swim (mean VO_2_swim) and 1-min-maximum run-induced VO_2_ (VO_2_run). Similar models, but without body mass as a covariate, were used to analyze the time of achieving VO_2_swim | |
| Saturated | **PROC** **Mixed** data=data method=reml alpha=**0.05** cl covtest ;  CLASS T S Drug ID L Trial SysNu LitNu ;  MODEL Y = T S Drug T*S T*Drug S*Drug T*S*Drug BM T*BM S*BM T*S*BM Drug*BM Drug*T*BM Drug*S*BM Drug*T*S*BM Trial SysNu LitNu LitS Age_t1 / DDFM=Satterthwaite ;  RANDOM L(T) S*L(T) Drug*L(T) Drug*S*L(T) BM*L(T) BM*S*L(T) BM*Drug*L(T) BM*Drug*S*L(T) ;  repeated Trial / type=cs subject=ID ;  **RUN** |
| Reduced | **PROC** **Mixed** data=data method=reml alpha=**0.05** cl covtest ;  CLASS T S Drug ID L Trial LitNu SysNu ;  MODEL Y = T S Drug T*S T*Drug BM BM*T Trial SysNu LitNu LitS Age_t1 / ddfm=satterthwaite solution cl ;  RANDOM L(T) S*L(T) Drug*L(T) BM*L(T) ;  REPEATED Trial / type=cs subject=ID ;  LSMEANS T / cl at Age_t1=**85** at LitS=**5.5** at BM=**14.6 diff** ;  LSMEANS T / cl at Age_t1=**85** at LitS=**5.5** at BM=**24.0 diff** ;  LSMEANS S Drug Trial T*Drug LitNu SysNu / cl at Age_t1=**85** at LitS=**5.5** at BM=**24.0** ;  **RUN** |
| The models concerning the proportional response (the ratio of VO2 after Drug to that after vehicle). | |
| Saturated | **PROC** **Mixed** data=data method=reml alpha=**0.05** cl covtest ;  CLASS T S ExType ID L LitNu ;  MODEL Y = T S ExType T*S T*ExType S*ExType T*S*ExType LitNu LitS / ddfm=satterthwaite ;  RANDOM L(T) S*L(T) ExType*L(T) ExType*S*L(T) ;  REPEATED ExType / type=cs subject=ID ;  **RUN** |
| Reduced | **PROC** **Mixed** data=data method=reml alpha=**0.05** cl covtest ;  CLASS T S ExType ID L LitNu ;  MODEL Y = T S ExType T*S T*ExType LitNu LitS / solution ddfm=satterthwaite ;  RANDOM L(T) Fam(L(T)) S*L(T) ExType*L(T) BM*L(T);  REPEATED ExType / type=cs subject=ID ;  LSMEANS T / cl LitS=**5.5** diff ;  LSMEANS S ExType LitNu T*ExType / cl at LitS=**5.5** ;  **RUN** |

**Table S2 Variables in the models presented in table S1.**

| Variable | description |
| --- | --- |
| Y | a dependent variable (e.g., VO2swim) |
| Main fixed effects: | |
| Drug | treatment (AM404 experiment: AM404 *vs* vehicle; Rimonabant experiment: Rimonabant *vs* vehicle) |
| Exp | Experiment type (AM404 *vs* Rimonabant) |
| ExType | Exercise type (swim *vs* run; a repeated measure factor) |
| S | Sex |
| T | line Type (selected *vs* control) |
| Trial | number of a Trial (1 – 3; a repeated measure factor) |
| Random effects: | |
| L(T) | replicate Line nested in line Type |
| Fam(L(T)) | Family nested in replicate Line |
| ID | the number of an individual |
| Covariates and cofactors: | |
| Age | age at the trial [days] |
| Age_t1 | age at the first trial [days] |
| BM | Body mass [g] |
| Date | date of selection swimming trial [Excel date numeric value] |
| LitS | litter Size |
| LitNu | consecutive Litter Number of a given female, in which the individual was born: 1, 2 or 3 |
| SysNu | Respirometric system number (1 *vs* 2) |

**Table S3 SAS Software commands, statements and options in the models presented in table S1.**

| Command | description |
| --- | --- |
| PROC Mixed | requests fitting mixed linear models |
| method | determines estimation method (restricted maximum likelihood; reml) |
| alpha | determines the confidence level for fixed effects (0.05) |
| cl | displays confidence limits |
| covtest | displays asymptotic standard errors and Wald tests for the covariance parameter estimates |
| CLASS | declares categorical variables |
| MODEL | the model statement (a single dependent variable and fixed effects) |
| solution | displays fixed-effects parameter estimates |
| ddfm | determines the method for computing denominator degrees of freedom (satterthwaite) |
| RANDOM | specifies random effects |
| REPEATED | a repeated measure statement |
| type | determines covariance structure (compound symmetry) |
| subject | determined the subject on which the repeated measure is operating (ID) |
| LSMEANS | computes least squares means for classification fixed effects |
| at | determines covariate value for computing least squares means |
| diff | requests differences of least squares means |
| adjust | performs multiple comparisons adjustments (Tukey, Dunnett) |
| SLICE | performes partitioned analysis |

**Table S4 Descriptive statistics of body mass and 1-min maximum rate of oxygen consumption (VO_2_) of all bank voles from selected A and unselected C lines from generation 22 (selection trial), and of subsamples of voles used in the two experiments with pharmacological manipulation (AM404 – an endocannabinoid reuptake inhibitor or Rimonabant – cannabinoid receptor CB1 antagonist)**  Arithmetic means ± standard deviations (SD) and the adjusted least square means ± standard errors (LSM±SE) from analyses of covariance (ANCOVA) are presented (LSMs of VO_2_ calculated for body mass of 24g). VO_2_swim – swim-induced VO2; VO_2_run – run-induced VO2.

| **Trial: trait** | | **Sex** | **Sample size** | | **Body mass [g]** | | **VO2 [mlO2/min]** | | | |
| --- | --- | --- | --- | --- | --- | --- | --- | --- | --- | --- |
|  | Treatment |  |  | | **mean ± SD** | | **mean ± SD** | | **LSM±SE** | |
|  |  |  | **C** | **A** | **C** | **A** | **C** | **A** | **C** | **A** |
| **Selection trial: VO_2_swim** | | | | |  |  |  |  |  |  |
|  | none | F | 85 | 278 | 20 ± 3.2 | 23 ± 3.1 | 3.2 ± 0.5 | 5.6 ± 0.6 | 3.5 ± 0.11 | 5.8 ± 0.09 |
|  |  | M | 93 | 330 | 24 ± 3.5 | 26 ± 3.5 | 3.5 ± 0.5 | 6.0 ± 0.7 | 3.5 ± 0.10 | 5.7 ± 0.09 |
| **AM404 experiment: VO_2_swim** | | | | |  |  |  |  |  |  |
|  | vehicle | F | 24 | 24 | 21 ± 2.7 | 23 ± 2.2 | 3.1 ± 0.59 | 5.7 ± 0.53 | 3.5 ± 0.15 | 5.8 ± 0.13 |
|  |  | M | 24 | 24 | 24 ± 3.5 | 28 ± 3.2 | 3.2 ± 0.50 | 6.1 ± 0.61 | 3.2 ± 0.13 | 5.8 ± 0.16 |
|  | AM404 | F | 24 | 25 | 20 ± 2.6 | 23 ± 2.2 | 3.0 ± 0.59 | 5.5 ± 0.65 | 3.5 ± 0.16 | 5.6 ± 0.13 |
|  |  | M | 21 | 24 | 25 ± 3.6 | 28 ± 3.5 | 3.1 ± 0.70 | 5.9 ± 0.64 | 3.0 ± 0.13 | 5.4 ± 0.16 |
| **AM404 experiment: VO_2_run** | | | | |  |  |  |  |  |  |
|  | vehicle | F | 23 | 23 | 20 ± 2.6 | 23 ± 2.4 | 4.0 ± 0.52 | 5.7 ± 0.84 | 4.4 ± 0.22 | 5.9 ± 0.18 |
|  |  | M | 22 | 23 | 24 ± 3.4 | 27 ± 3.4 | 4.5 ± 0.65 | 6.2 ± 0.82 | 4.5 ± 0.19 | 5.7 ± 0.20 |
|  | AM404 | F | 24 | 23 | 20 ± 2.5 | 23 ± 2.5 | 4.0 ± 0.55 | 5.6 ± 0.96 | 4.3 ± 0.22 | 5.9 ± 0.18 |
|  |  | M | 22 | 23 | 24 ± 3.5 | 27 ± 3.2 | 4.3 ± 0.55 | 6.0 ± 0.75 | 4.3 ± 0.18 | 5.4 ± 0.20 |
| **Rimonabant experiment: VO_2_swim** | | | | |  |  |  |  |  |  |
|  | vehicle | F | 24 | 24 | 21 ± 3.9 | 25 ± 2.7 | 3.1 ± 0.78 | 5.8 ± 0.53 | 3.5 ± 0.16 | 5.8 ± 0.15 |
|  |  | M | 23 | 24 | 24 ± 3.7 | 27 ± 3.0 | 3.5 ± 0.74 | 6.1 ± 0.76 | 3.5 ± 0.16 | 5.8 ± 0.17 |
|  | Rimonabant | F | 24 | 24 | 20 ± 4.0 | 24 ± 2.4 | 3.3± 0.69 | 5.7 ± 0.57 | 3.7 ± 0.16 | 5.8 ± 0.15 |
|  |  | M | 23 | 24 | 24 ± 4.1 | 27 ± 2.5 | 3.4 ± 0.69 | 6.1 ± 0.72 | 3.4 ± 0.16 | 5.8 ± 0.17 |
| **Rimonabant experiment: VO_2_run** | | | | |  |  |  |  |  |  |
|  | vehicle | F | 24 | 24 | 20 ± 3.7 | 24 ± 2.3 | 4.2 ± 0.51 | 5.8 ± 0.60 | 4.6 ± 0.20 | 5.8 ± 0.19 |
|  |  | M | 22 | 23 | 24 ± 4.2 | 27 ± 3.1 | 4.5 ± 0.47 | 6.2 ± 1.06 | 4.5 ± 0.20 | 5.6 ± 0.21 |
|  | Rimonabant | F | 24 | 23 | 20 ± 3.7 | 25 ± 2.4 | 4.0 ± 0.72 | 5.9 ± 0.50 | 4.5 ± 0.20 | 5.8 ± 0.19 |
|  |  | M | 21 | 24 | 24 ± 4.4 | 27 ± 3.1 | 4.5 ± 0.56 | 6.1 ± 1.03 | 4.5 ± 0.20 | 5.7 ± 0.21 |
|  |  |  |  |  |  |  |  |  |  |  |

**Table S5 The effects of body mass, line type (selection direction) and their interaction on the rate of oxygen consumption (VO_2_) in all bank voles from generation 22 of the selection experiment (selection trial) and in the two experiments with pharmacological manipulation (AM404 – an endocannabinoid reuptake inhibitor or Rimonabant – cannabinoid receptor CB1 antagonist)** Results of the analyses of covariance (ANCOVA) for the line Type effect provides a test of significance of the difference between adjusted means of A and C lines at the minimum (16.4g in AM404: VO_2_run and 14.6g in the other analyses) and mean (24g) body mass (BM; see Methods for the rationale of the analysis). Body mass describes the regression slope for C lines and the line Type×Body Mass interaction describes the difference in regression slopes between the selection groups. VO_2_swim – swim-induced VO_2_; VO_2_run – run-induced VO_2_; meanVO_2_swim – whole-trial mean VO_2_swim; Df – degrees of freedom; Ndf – numerator df; Ddf – denominator df; SE – standard error.

| Trial: Trait | Body mass | | line Type×Body Mass | | line Type | |
| --- | --- | --- | --- | --- | --- | --- |
|  |  | |  | | at minimum  BM | at mean BM |
|  | slopes for C lines±SE  [mlO_2_/(min×g)] | t value (df)  p value | Difference in slopes A - C lines±SE  [mlO_2_/(min×g)] | t value (df)  p value | t value  (df)  p value | t value  (df)  p value |
| Selection trial: VO_2_swim | 0.08±0.013 | 5.63 (228)  <0.0001 | 0.05±0.015 | 3.58 (191)  0.0004 | 11.5 (86)  <0.0001 | 18.9 (8)  <0.0001 |
| Exercise Type: VO_2_swim and VO_2_run combined | 0.13±0.017 | 7.37 (104) <0.0001 | 0.02±0.025 | 0.86 (127)  0.39 | 5.82 (76)  <0.0001 | 8.43 (6)  0.0001 |
| AM404: VO_2_swim | 0.12±0.025 | 4.95 (51) <0.0001 | -0.01±0.036 | 0.43 (76)  0.66 | 6.94 (81)  <0.0001 | 15.6 (8)  <0.0001 |
| AM404: meanVO_2_swim | 0.09±0.027 | 3.41 (62) 0.001 | -0.01±0.040 | 0.25 (91)  0.80 | 13.6 (8)  <0.0001 | 6.17 (95)  <0.0001 |
| AM404: VO_2_run | 0.11±0.033 | 3.25 (56) 0.002 | 0.06±0.045 | 1.46 (68)  0.15 | 2.31 (54)  0.02 | 5.9 (7)  0.0005 |
| Rimonabant: VO_2_swim | 0.09±0.021 | 4.38 (104)  <0.0001 | -0.005±0.0317 | 0.16 (125)  0.87 | 6.46 (105)  <0.0001 | 14.2 (21)  <0.0001 |
| Rimonabant: meanVO_2_swim | 0.08±0.022 | 3.65 (69)  0.0005 | 0.007±0.0331 | 0.22 (113)  0.82 | 5.92 (118)  <0.0001 | 12.6 (9)  <0.0001 |
| Rimonabant: VO_2_run | 0.12±0.017 | 7.20 (68) <0.0001 | 0.04±0.027 | 1.49 (76)  0.14 | 2.38 (39)  0.02 | 5.76 (7)  0.0006 |

**Table S6 The effects of additional factors used in ANCOVA models on the main traits measured in all bank voles from generation 22 of the selection experiment (selection trial) and the two experiments with pharmacological manipulation (Exercise Type; pulled data from AM404 and Rimonabant)** VO_2_swim, VO_2_run – 1‑min maximum swim- or run-induced rate of oxygen consumption; F or t statistics; df – degrees of freedom; Ndf –numerator df; Ddf – denominator df; LSM(95%CL) – Least squares mean with 95% conﬁdence limits. For the main effects see Table 1 and Results section in the main text.

| Trial: Trait | Sex | Line TypexSex | Litter Size | Age | Litter Number | |
| --- | --- | --- | --- | --- | --- | --- |
|  | F (Ndf,Ddf)  p value | F (Ndf,Ddf)  p value | t (df)  p value  slope±SE | t (df)  p value  slope±SE | F (Ndf, Ddf)  p value | LSM(95%CL)  1^st^  2^nd^  3^rd^ |
| Selection trial:  Body mass | 101.9 (1,7)  <0.0001 | 1.09 (1,7)  0.33 | 3.22 (637)  0.001  -0.3±0.08 | 3.75 (760)  0.0002  0.12±0.031 | 19.11 (2,741)  <0.0001 | 22.5(20.9-24.2)  23.6(21.9-25.2)  24.5(22.9-26.2) |
| Selection trial:  VO_2_swim | 0.43 (1,14)  0.52 | 0.06 (1,14)  0.81 | 2.19 (519)  0.029  -0.03±0.015 | 0.41 (761)  0.68  0.002±0.0057 | 2.36 (2,747)  0.09 | 4.62(4.81-4.77)  4.65(4.51-4.80)  4.52(4.35-4.69) |
| Exercise Type:  VO_2_swim and VO_2_run combined* | 2.52 (1,187)  0.11 | 0.06 (1,185)  0.81 | 0.32 (197)  0.75  -0.007±0.022 | 0.95 (113)  0.35  0.01±0.016 | 0.62 (2,161)  0.54 | 4.91(4.66-5.16)  4.92(4.67-5.17)  4.83(4.58-5.09) |

*Exercise Type effect: F_1,7_=67.1, p<0.0001, Line Type×Exercise Type: F_1,7_=26.9, p=0.0011

**Table S7 The effects of additional factors used in ANCOVA models on the main traits measured in the experiment with endocannabinoids reuptake inhibitor (AM404)** VO_2_swim, VO_2_run – 1-min maximum swim- or run-induced rate of oxygen consumption; time at VO_2_swim - the time of achieving the 1-min VO2swim; meanVO2swim – whole-trial mean VO_2_swim; proportional response – the ratio of VO_2_swim or VO_2_run achieved after drug to the one after vehicle; F or t statistics; df – degrees of freedom; Ndf – numerator df; Ddf – denominator df; LSM(95%CL) – Least squares mean with 95% conﬁdence limits. For the main effects see Tables 1, 2 and Results section in the main text.

| Dependent variable | Sex | Line TypexSex | Trial Number | | Litter Size | Age | Litter Number | |
| --- | --- | --- | --- | --- | --- | --- | --- | --- |
|  | F  (Ndf,Ddf)  p value | F  (Ndf,Ddf)  p value | F  (Ndf,Ddf)  p value | LSM(95%CL)  1^st^  2^nd^ | t (df)  p value  slope±SE | t (df)  p value  slope±SE | F  (Ndf, Ddf)  p value | LSM(95%CL)  1^st^  2^nd^  3^rd^ |
| VO_2_swim | 3.72 (1,97)  0.06 | 1.35 (1,98)  0.25 | 1.69 (1,90)  0.20 | 4.45(4.27-4.63)  4.51(4.33-4.68) | 1.33 (83)  0.18  0.05±0.039 | 0.49 (86)  0.62  -0.012±0.0236 | 0.50 (2,81)  0.61 | 4.54(4.32-4.76)  4.48(4.28-4.67)  4.41(4.18-4.64) |
| time at VO_2_swim | 2.56 (1,88)  0.11 | 0.22 (1,88)  0.74 | 1.09 (1,93)  0.30 | 689(642-735)  657(610-705) | 1.56 (86)  0.12  23±14.8 | 2.17 (87)  0.03  -19±8.7 | 2.37 (2,87)  0.10 | 675(610-740)  723(665-782)  621(548-693) |
| mean VO_2_swim | 1.58 (1,101)  0.21 | 1.23 (1,104)  0.27 | 0.07 (1,89)  0.79 | 3.90(3.70-4.10)  3.91(3.71-4.12) | 0.57 (84)  0.57  0.026±0.0463 | 0.56 (86)  0.58  -0.015±0.0274 | 0.20 (2,81)  0.82 | 3.96(3.71-4.21)  3.89(3.66-4.11)  3.88(3.62-4.15) |
| VO_2_run | 1.56 (1,78)  0.22 | 0.90 (1,79)  0.34 | 0.01 (1,81)  0.92 | 5.06(4.79-5.32)  5.05(4.79-5.31) | 0.29 (71)  0.77  0.013±0.044 | 0.23 (74)  0.82  0.006±0.0269 | 3.16 (2,72)  0.05 | 5.04(4.75-5.33)  5.23(4.95-5.51)  4.88(4.58-5.19) |
| proportional response | 0.83 (1,83)  0.36 | 0.11 (1,82)  0.74 |  |  | 0.05 (72)  0.96  0.0003±0.00675 |  | 0.87 (2,82)  0.42 | 0.97(0.94-1.01)  0.99(0.96-1.02)  0.97(0.93-1.01) |

**Table S8 The effects of additional factors used in ANCOVA models on the main traits measured in the experiment with endocannabinoid receptor CB1 antagonist (Rimonabant)** VO_2_swim, VO_2_run – 1-min maximum swim- or run-induced rate of oxygen consumption; time at VO_2_swim - the time of achieving the 1-min VO2swim; meanVO_2_swim – whole-trial mean VO_2_swim; proportional response – the ratio of VO_2_swim or VO_2_run achieved after drug to the one after vehicle; F or t statistics; df – degrees of freedom; Ndf – numerator df; Ddf – denominator df; LSM(95%CL) – Least squares mean with 95% conﬁdence limits. For the main effects see Tables 1, 2 and Results section in the main text.

| Dependent variable | Sex | Line TypexSex | Trial Number | | Litter Size | Age | Litter Number | |
| --- | --- | --- | --- | --- | --- | --- | --- | --- |
|  | F  (Ndf,Ddf)  p value | F  (Ndf,Ddf)  p value | F  (Ndf,Ddf)  p value | LSM(95%CL)  1^st^  2^nd^ | t (df)  p value  slope±SE | t (df)  p value  slope±SE | F  (Ndf, Ddf)  p value | LSM(95%CL)  1^st^  2^nd^  3^rd^ |
| VO_2_swim | 0.02 (1,13)  0.88 | 0.23 (1,14)  0.64 | 1.07 (1,89)  0.30 | 4.68(4.51-4.85)  4.64(4.46-4.81) | 2.70 (82)  0.008  -0.096±0.0355 | 1.66 (77)  0.10  0.045±0.0269 | 1.59 (2,73)  0.21 | 4.59(4.38-4.81)  4.59(4.36-4.82)  4.80(4.57-5.02) |
| time at VO_2_swim | 3.04 (1,81)  0.08 | 0.53 (1,82)  0.47 | 0.63 (1,92)  0.43 | 618(544-692)  593(519-667) | 0. 05 (87)  0.62  -6.4±12.81 | 0.03 (84)  0.97  0.3±9.71 | 0.33 (2,81)  0.72 | 584(503-665)  622(536-708)  610(526-694) |
| mean VO_2_swim | 0.00 (1,132)  0.98 | 0.05 (1,88)  0.83 | 0.25 (1,91)  0.62 | 4.12(3.92-4.32)  4.10(3.90-4.31) | 2.54 (84)  0.01  -0.09±0.037 | 1.54 (81)  0.13  0.04±0.028 | 1.40 (2,81)  0.25 | 4.10(3.86-4.34)  4.00(3.75-4.25)  4.23(4.00-4.48) |
| VO_2_run | 0.29 (1,9)  0.60 | 0.07 (1,10)  0.79 | 0.53 (1,72)  0.47 | 5.17(4.92-5.42)  5.12(4.87-5.36) | 0.92 (72)  0.36  0.03±0.029 | 0.58 (63)  0.56  0.01±0.021 | 7.27 (2,66)  0.001 | 5.26(5.00-5.52)  5.26(5.00-5.53)  4.90(4.64-5.16) |
| proportional response | 0.38 (1,90)  0.54 | 0.58 (1,87)  0.45 |  |  | 0.59 (107)  0.55  -0.003±0.0056 |  | 1.13 (2,87)  0.33 | 1.00(0.95-1.05)  1.02(0.96-1.07)  0.99(0.93-1.04) |

**Table S9 The t statistics and adjusted p values from Tukey comparisons Line Type × Exercise type × Sex in the analysis of the proportional response to endocannabinoid receptor CB1 antagonist (Rimonabant; the ratio of VO_2_swim or VO_2_run achieved after Rimonabant to the one after vehicle)** t (df) ‑ t statistics and degrees of freedom

| **group 1** | | | ***vs*** | **group 2** | | | **t (df)** | **p value** |
| --- | --- | --- | --- | --- | --- | --- | --- | --- |
| **Line Type** | **Exercise**  **Type** | **Sex** |  | **Line Type** | **Exercise**  **Type** | **Sex** |  |  |
| C | swim | 0 |  | C | swim | 1 | 3.28 (123) | 0.03 |
| C | run | 0 |  | C | run | 1 | 1.24(130) | 0.92 |
| C | run | 0 |  | C | swim | 0 | 2.93 (14) | 0.08 |
| C | run | 1 |  | C | swim | 1 | 1.5 (19) | 0.80 |
| A | swim | 0 |  | A | swim | 1 | 0.55 (123) | 1 |
| A | run | 0 |  | A | run | 1 | 0.78(133) | 1 |
| A | run | 0 |  | A | swim | 0 | 1.32 (17) | 0.89 |
| A | run | 1 |  | A | swim | 1 | 0.57 (17) | 1 |
| A | swim | 0 |  | C | swim | 0 | 1.36 (10) | 0.87 |
| A | swim | 1 |  | C | swim | 1 | 0.92 (10) | 0.98 |
| A | run | 0 |  | C | run | 0 | 1.26 (14) | 0.91 |
| A | run | 1 |  | C | run | 1 | 0.19 (16) | 1 |
|  |  |  |  |  |  |  |  |  |

**Table S10** **The variance components estimates ± standard error (SE) and likelihood ratio test (LRT) statistics for the significance of the random effects** (χ^2^ test with 1 degree of freedom; χ^2^ = -2log(reduced/full model and p values). L(T) - replicate Line nested in line Type; Fam(L(T)) - Family nested in replicate Line; ID - the number of an individual; Line interactions with fixed factors: S – Sex, drug – treatment, ExType – exercise type, BM – body mass. Value 0 means that the variance component estimate was fixed to 0 and the LRT was not performed as it would be nonsensical (NA – not applicable).

| Analysis | L(T) | Fam(L(T)) | S*L(T) | | drug*L(T) | ExType*L(T) | BM*L(T) | ID | residual |
| --- | --- | --- | --- | --- | --- | --- | --- | --- | --- |
| Dependent variable | | | |  | | | |  |  |
| Selection Trial |  |  |  | |  |  |  |  |  |
| Body mass |  |  |  | |  |  |  |  |  |
| estimate±SE | 3±2 | 2±0.4 | 0.2±0.3 | |  |  |  |  | 6.2±0.3 |
| χ^2^; p value | 63.5; <0.001 | 96.9; <0.001 | 4.9; 0.03 | |  |  |  |  |  |
| VO_2_swim |  |  |  | |  |  |  |  |  |
| estimate±SE | 0 | 0.04±0.01 | 0.007±0.006 | |  |  | 0.00003±0.00003 |  | 0.2±0.01 |
| χ^2^; p value | NA | 35; <0.001 | 5.3; 0.02 | |  |  | 3.3; 0.07 |  |  |
| Exercise Type |  |  |  | |  |  |  |  |  |
| VO_2_swim and VO_2_run | |  |  | |  |  |  |  |  |
| estimate±SE | 0 | 0.05±0.03 | 0 | |  | 0.01±0.01 | 0.0001±0.0001 | 0.02±0.03 | 0.02±0.03 |
| χ^2^; p value | NA | 4; 0.04 | NA | |  | 3.2; 0.07 | 6.7; 0.01 | 0.6; 0.44 |  |

| Analysis | L(T) | Fam(L(T)) | S*L(T) | | drug*L(T) | ExType*L(T) | BM*L(T) | ID | residual |
| --- | --- | --- | --- | --- | --- | --- | --- | --- | --- |
| Dependent variable | | | |  | | | |  |  |
| AM404 experiment | | | | | | | |  |  |
| VO_2_swim |  |  |  | |  |  |  |  |  |
| estimate±SE | 0 |  | 0 | | 0.006±0.008 |  | 0.00003±0.00004 | 0.2±0.03; | 0.08±0.01 |
| χ^2^; p value | NA |  | NA | | 1.3; 0.25 |  | 1.1; 0.29 | 47; <0.001 |  |
| time at VO_2_swim | |  |  | |  |  |  |  |  |
| estimate±SE | 0 |  | 0 | | 0 |  |  | 9703±5711 | 43100±6361 |
| χ^2^; p value | NA |  | NA | | NA |  |  | 3.0; 0.08 |  |
| mean VO_2_swim | |  |  | |  |  |  |  |  |
| estimate±SE | 0 |  | 0 | | 0.007±0.007 |  | 0.00004±0.00005 | 0.2±0.05; | 0.06±0.01 |
| χ^2^; p value | NA |  | NA | | 2.3; 0.13 |  | 1.5; 0.22 | 86.5; <0.001 |  |
| VO_2_run |  |  |  | |  |  |  |  |  |
| estimate±SE | 0 |  | 0 | | 0 |  | 0.0001±0.0001 | 0.1±0.04 | 0.2±0.03 |
| χ^2^; p value | NA |  | NA | | NA |  | 8.1; 0.004 | 12.6; <0.001 |  |
| proportional response | |  |  | |  |  |  |  |  |
| estimate±SE | 0 |  | 0 | |  | 0.0001±0.0005 |  | 0.0003±0.001 | 0.01±0.002 |
| χ^2^; p value | NA |  | NA | |  | 0.1; 0.75 |  | 0.1; 0.75 |  |

| Analysis | L(T) | Fam(L(T)) | S*L(T) | | drug*L(T) | ExType*L(T) | BM*L(T) | ID | residual |
| --- | --- | --- | --- | --- | --- | --- | --- | --- | --- |
| Dependent variable | | | |  | | | |  |  |
| Rimonabant experiment | | | | | | | |  |  |
| VO_2_swim |  |  |  | |  |  |  |  |  |
| estimate±SE | 0 |  | 0.01±0.03 | | 0.02±0.02 |  | 0 | 0.2±0.04 | 0.09±0.01 |
| χ^2^; p value | NA |  | 0.4; 0.52 | | 10.8; 0.001 |  | NA | 49.2; <0.001 |  |
| time at VO_2_swim | |  |  | |  |  |  |  |  |
| estimate±SE | 4e3±4e3 |  | 0 | | 0 |  |  | 8e3±6e3 | 5e4±0.7e4 |
| χ^2^; p value | 10.8; 0.001 |  | NA | | NA |  |  | 1.9; 0.17 |  |
| mean VO_2_swim | |  |  | |  |  |  |  |  |
| estimate±SE | 0 |  | 0 | | 0.02±0.01 |  | 0.00003±0.00005 | 0.2±0.04 | 0.08±0.01 |
| χ^2^; p value | NA |  | NA | | 8; 0.005 |  | 0.8; 0.37 | 62.3; <0.001 |  |
| VO_2_run |  |  |  | |  |  |  |  |  |
| estimate±SE | 0.04±0.05 |  | 0.01±0.02 | | 0.04±0.03 |  | 0 | 0.02±0.03; | 0.2±0.03 |
| χ^2^; p value | 20.7; <0.001 |  | 0.7; 0.40 | | 5; 0.02 |  | NA | 0.7; 0.40 |  |
| proportional response* | |  |  | |  |  |  |  |  |
| estimate±SE | 0.0028±0.00200 |  | 0 | |  | 0.0002±0.00071 |  | 0.001±0.0012 | 0.008±0.0014 |
| χ^2^; p value | 16.0; <0.001 |  | NA | |  | 0.1; 0.75 |  | 1.3; 0.25 |  |
| * variance component estimate for S*ExType*L(T) interaction was fixed to zero and therefore the LRT was not performed. | | | | | | | |  |  |
